# Remnant Cholesterol, a Valuable Biomarker for Assessing Arteriosclerosis and Cardiovascular Risk: A Systematic Review

**DOI:** 10.7759/cureus.44202

**Published:** 2023-08-27

**Authors:** Xiang Chen, Li-Hua Li

**Affiliations:** 1 Gerontology, The First Affiliated Hospital of Dali University, Dali, CHN

**Keywords:** cardiovascular disease, triglyceride rich lipoproteins, atherosclerosis (as), arteriosclerosis, remnant cholesterol

## Abstract

Arteriosclerosis, as the foundation for the development of cardiovascular diseases, is influenced by disturbances in lipid metabolism. Extensive research has consistently shown a correlation between conventional lipid parameters, arteriosclerosis, and cardiovascular diseases. Guidelines highlight the importance of targeting low-density lipoprotein cholesterol (LDL-C) for primary and secondary prevention of cardiovascular diseases, with reducing LDL-C remaining the primary lipid-lowering strategy. However, even when LDL-C is lowered to optimal levels, there is a residual risk of cardiovascular disease. Recent findings have brought attention to remnant cholesterol (RC) as a significant factor contributing to this residual risk. The close association between RC, arteriosclerosis, and cardiovascular diseases presents exciting opportunities for lifestyle interventions and medical treatments to control and lower RC levels, offering new targets for preventing and managing related cardiovascular conditions. Our systematic review sheds light on the importance of considering RC as a valuable biomarker in assessing arteriosclerosis and its potential impact on cardiovascular health. By understanding the link between remnant cholesterol and arteriosclerosis, researchers and healthcare professionals can develop targeted interventions to mitigate cardiovascular risks, thus improving public health outcomes and reducing the economic burden associated with cardiovascular diseases.

## Introduction and background

Arteriosclerosis is a critical risk factor for cardiovascular disease (CVD) [1], and lipid metabolism disorders are key contributors to its development [2,3]. Despite achieving optimal levels of low-density lipoprotein cholesterol (LDL-C), some patients still experience residual risk of CVD. Remnant cholesterol (RC) has emerged as a potential cause of this residual risk [4]. The relationship between RC, arteriosclerosis, and CVD is well-established [2,3,5,6]. Lifestyle interventions and drug therapies have proven effective in reducing RC levels, providing a new therapeutic approach for the prevention and treatment of related CVD. An in-depth understanding of the link between RC and arteriosclerosis is of great clinical significance for the diagnosis, treatment, and prognosis of CVD. This review aims to provide a comprehensive analysis of the current research progress on the relationship between RC and arteriosclerosis, identify gaps in knowledge, and highlight areas for future research.

## Review

Arteriosclerosis

Definition, Mechanisms, and Measurement Indices of Arteriosclerosis

Arteriosclerosis is a degenerative process of the extracellular matrix of the arterial medial membrane, characterized by the thickening and hardening of the arterial wall due to structural and functional changes, resulting in a loss of elasticity and narrowing of the lumen [1,2,7]. Atherosclerosis is primarily characterized by the formation of lipid-rich plaques in the arterial intima but can also sometimes present as aneurysms or ectasia [1]. In contrast, arteriosclerosis is associated with impaired elastin/collagen ratio, reactive oxygen-induced inflammation, vascular calcification, vascular smooth muscle cell sclerosis, and endothelial dysfunction [8]. It often coexists with atherosclerosis and may represent the cause rather than the effect of atherosclerosis [9]. Risk factors for arteriosclerosis include age, hypertension, diabetes, obesity, smoking, and genetic factors. Arteriosclerosis has an independent predictive value for cardiovascular events as an intermediate endpoint beyond traditional risk factors [10,11]. The presence of early arteriosclerosis can be proved by the abnormal results of the assessment of pulse wave velocity (PWV), augmentation index, and central blood pressure [12,13].

The Link Between Arteriosclerosis and Diseases

Arteriosclerosis is closely associated with hypertension, diabetes, chronic kidney disease (CKD), and CVD and can be used as a predictor of these diseases [9,14,15]. Arteriosclerosis is an important risk factor for CVD and is prevalent in CVD patients. Assessing the degree of arteriosclerosis is clinically significant for primary and secondary prevention in patients with CVD [13]. For instance, brachial-ankle pulse wave velocity is an independent predictor of CVD risk in healthy individuals. Furthermore, its measurement improves the effectiveness of predicting the risk of CVD development [16]. Aortic stiffness and brachial-ankle pulse wave velocity are predictors of cardiovascular events in patients with stable angina pectoris and non-ST-elevation myocardial infarction, and can significantly predict stroke death [17-19].

Increasing age and elevated blood pressure are major factors in arteriosclerosis. The use of antihypertensive drugs such as angiotensin-converting enzyme inhibitors (ACEIs), angiotensin receptor blockers (ARBs), and calcium channel blockers (CCBs) has been shown to improve arteriosclerosis, while lifestyle changes such as smoking cessation, weight loss, and physical exercise also have a moderating effect [11]. However, relying solely on these methods is insufficient to achieve the purpose of reducing cardiovascular events. Therefore, early identification of arteriosclerosis and its risk factors, as well as early intervention to prevent elevated pulse wave velocity, are particularly important for reducing the risk of CVD.

Blood lipid profile and arteriosclerosis

The Relationship Between Conventional Lipid Parameters and Arteriosclerosis

Recent studies have shown that there is a correlation between lipid parameters and arteriosclerosis [2,3,6]. A cross-sectional study of the general population in Japan found that high-density lipoprotein (HDL-C) levels were negatively correlated with brachial-ankle pulse wave velocity, while total cholesterol (TC), triglyceride (TG), and LDL-C levels were positively correlated with brachial-ankle pulse wave velocity. However, after adjusting for various factors, only TG levels remained positively correlated with brachial-ankle pulse wave velocity [7]. A study found that high TG/HDL-C was an important risk factor for arteriosclerosis in a healthy Chinese adult sample [20]. Moreover, the Chinese Stroke Primary Prevention Trial (CSPPT) demonstrated an association between lipid profiles and arteriosclerosis in adult patients with essential hypertension in China [21]. These findings highlight that lipid metabolism disorders are a crucial factor in the development of arteriosclerosis and CVD.

Understanding RC: Definition, Metabolism, Measurement, and Calculation

**Figure 1:**
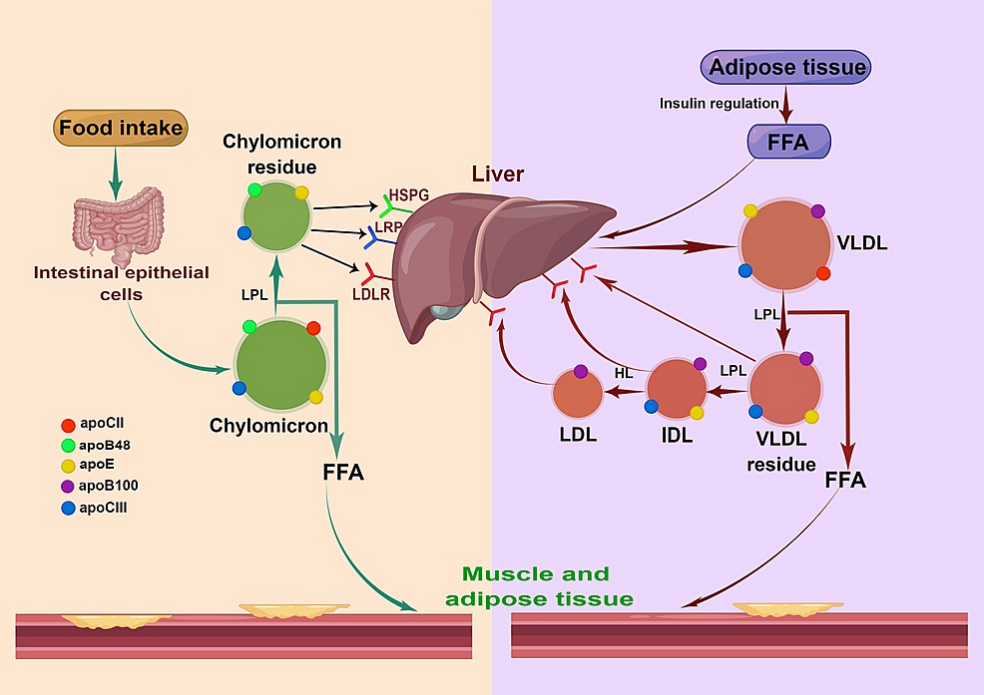
Metabolic pathways of TGRL production The figure illustrates the metabolic pathways involved in the synthesis of TGRLs. Chylomicrons and VLDL are hydrolyzed to produce TGRL remnants, which can increase remnant cholesterol levels. TGRL: Triglyceride-rich lipoprotein; HSPG: Heparan sulfate proteoglycan; LDL: Low-density lipoprotein; LDLR: Low-density lipoprotein receptor; LRP: LDL receptor-like protein; LPL: Lipoprotein lipase; HL: Hepatic lipase; FFA: Free fatty acid This diagram has been created by the authors using Figdraw.

Remnant cholesterol is a cholesterol component of triglyceride-rich lipoproteins (TGRLs) that consist of chylomicron residues in the non-fasting state, intermediate-density lipoprotein (IDL) in the fasting state, and very low-density lipoprotein (VLDL) [3,22]. Elevated plasma triglycerides and TGRLs are markers of elevated RC, of which TGRLs are derived from two pathways in humans: the endogenous pathway synthesized in the liver and the exogenous pathway synthesized and processed in the gastrointestinal system [23]. In the endogenous pathway, VLDL is produced in the endoplasmic reticulum of hepatocytes by free fatty acids (FFA) derived from the circulation and newly synthesized in the liver. During the secretion process, various apolipoproteins are added to the surface of VLDL particles. The VLDL secreted into the plasma is hydrolyzed by lipoprotein lipase (LPL) to produce FFA, VLDL residues, IDL particles, etc. The IDL particles are further catabolized into LDL and lipolytic products by hepatic lipase (HL). In the exogenous pathway, dietary fats are absorbed by intestinal cells and incorporated into chylomicrons containing apoB48, which are secreted into the lymphatic system and enter the circulation to obtain apolipoproteins such as apoC and apoE. The LPL then hydrolyzes chylomicrons to produce chylomicron residual particles and FFA. These endogenous and exogenous residues are eliminated from circulation by binding to liver surface LDL receptors, LDL receptor-like proteins (LRP), and heparan sulfate proteoglycan receptors (HSPG) (Figure 1) [4,23].

Chylomicrons and VLDL are lipolyzed to produce TGRL remnants, which can increase RC levels. The secretion of chylomicrons is mainly regulated by food intake, while the secretion of VLDL is controlled by insulin [4]. Currently, there is no established normal range for RC, and previous studies have often used the calculation formula RC = TC-LDL-C-HDL-C to calculate RC values. In addition, RC can also be directly measured using various methods such as ultracentrifugation, nuclear magnetic resonance spectroscopy, and direct automated measurement [3,23,24]. However, the clinical applicability of these direct measurement methods is limited and costly, so the calculation method is often used in research.

Remnant cholesterol and CVD

Remnant Cholesterol and Atherosclerotic CVD

Atherosclerotic CVD poses a significant threat to human life, with high incidence and mortality rates worldwide. Elevated LDL-C is a well-established risk factor for atherosclerotic CVD, making the reduction of LDL-C levels a crucial target for primary and secondary prevention. Statins, bile acid sequestrants, and other lipid-lowering drugs have been shown to significantly decrease the incidence of atherosclerotic CVD. However, even when LDL-C levels are optimally reduced, residual risks for CVD persist, such as elevated TG levels, RC, and lipoprotein (a) (Lp(a)).

Increased TG levels correspond to a higher number of TGRLs in circulation, which are cholesterol-rich and can elevate RC levels after hydrolysis. Research on patients with severe hypertriglyceridemia due to LPL-related gene mutations has shown that these individuals do not develop atherosclerosis in the absence of other cardiovascular risk factors [25]. Moreover, evidence suggests that TGRLs are more likely to penetrate the arterial intima during lipid circulation, entering the intima space where they are engulfed by macrophages. While macrophages can metabolize TG, they cannot metabolize RC, leading to its accumulation in the arterial wall and the development of atherosclerosis [23]. Although the role of RC in atherosclerotic plaque formation remains a topic of debate, recent studies have identified RC as the primary factor for the significant reduction in CVD risk among patients with lowered LDL-C levels [3,24].

A prospective study conducted by Duran et al. examined the relationship between RC and concentrations of small dense LDL cholesterol (sdLDLC), as well as CVD outcomes, including myocardial infarction (MI), ischemic stroke (IS), and peripheral artery disease (PAD). The study found that RC was strongly associated with future MI and PAD events [26]. Similarly, a Chinese study involving 409 patients (273 with coronary heart disease (CHD) and 136 non-CHD controls) demonstrated that RC is an independent predictor of CHD [27].

Research conducted by Castañer et al. revealed that both TG and RC are associated with atherosclerotic CVD outcomes, independent of other risk factors [28]. A study of the Copenhagen general population established a causal relationship between non-fasting RC elevation, low-grade inflammation, and an increased risk of ischemic heart disease (IHD) [29]. Furthermore, a prospective study of the Danish general population showed that reducing RC levels by 32 mg/dl (0.83 mmol/l) could decrease recurrent major adverse cardiovascular events (MACE) by 20% [30]. Collectively, these studies highlight the critical role of RC in the development of atherosclerotic CVD.

Mechanisms of RC in Promoting Atherosclerosis

Remnant cholesterol plays a critical role in promoting the formation and development of atherosclerosis through adhesion and pro-inflammatory effects, leading to the occurrence of related diseases [31]. First, RC can increase the production of reactive oxygen species, leading to endothelial dysfunction (ED). Additionally, RC can induce the apoptosis of endothelial cells by increasing the secretion of tumor necrosis factor-alpha (TNF-α) and interleukin (IL)-1β, further promoting the formation of atherosclerosis. Impaired endothelium-dependent vasodilation and increased oxidative stress may also play a role in promoting ED [23,32,33]. Second, RC can cause white blood cell migration and promote inflammation by producing cytokines and pro-atherosclerotic factors, leading to the formation of atherosclerosis. Finally, RC enhances platelet activity and aggregation by assembling the thromboplastin complex, upregulating the plasminogen activator inhibitor-1 gene, and expressing the plasminogen activator inhibitor-1 antigen, which results in thrombosis and atherosclerosis [34]. These mechanisms suggest that RC can directly contribute to the development of atherosclerosis by promoting ED, inflammation, and thrombosis.

Remnant Cholesterol and Arteriosclerosis

Lipid metabolism disorder is a crucial factor in arteriosclerosis, and RC is an essential indicator of lipid metabolism. Numerous studies have shown that RC levels are closely related to the occurrence of arteriosclerosis. A study by Wang et al. in a population without CVD found that participants with arteriosclerosis had significantly higher RC levels than those without arteriosclerosis [3]. The discriminative ability of RC in predicting arteriosclerosis was significantly higher than that of other prediction parameters such as HDL-C, plasma atherogenic index (AIP), atherogenic index (AI), and TG/HDL-C. This study indicates that RC is independently associated with the risk of arteriosclerosis in the general population without CVD [3]. Another study confirmed the strong and independent association between RC and brachial-ankle pulse wave velocity [6]. Qian et al.'s study showed that there is a linear correlation between RC levels, abnormal average carotid intima-media thickness (cIMT), and abnormal maximum cIMT, which still exists even under optimal LDL-C levels [35]. In a large population study of 1934 participants, researchers used the heart-ankle vascular index (CAVI) as an indicator of arteriosclerosis to investigate the relationship between TG levels and arteriosclerosis. The study found that even after adjusting for confounding factors such as age, gender, statin therapy, and smoking habits, high TG levels were still related to CAVI [36]. Plasma TG levels correspond to TGRLs and their residuals, which include RC. These studies indirectly indicate that RC is associated with arteriosclerosis. Taken together, these findings suggest that RC is an important indicator for predicting the risk of arteriosclerosis and is closely related to subclinical atherosclerosis even under optimal LDL-C levels.

Mechanisms of Remnant Cholesterol in Promoting Arteriosclerosis

**Figure 2:**
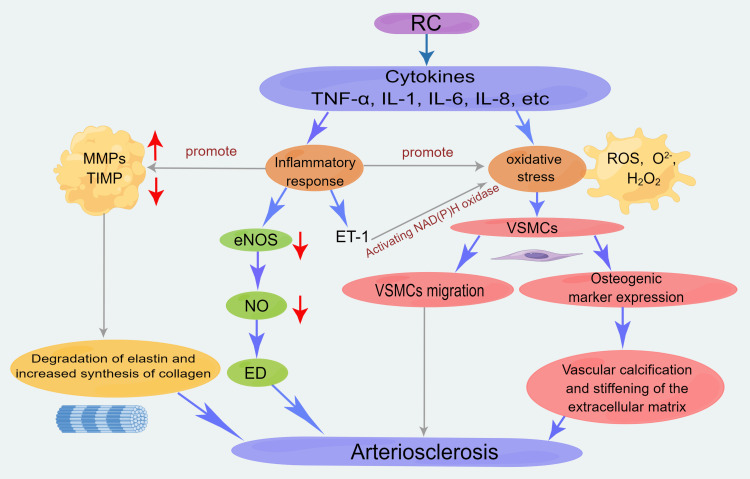
Potential mechanisms by which RC contributes to arteriosclerosis RC: Remnant cholesterol; MMP: Matrix metalloproteinase; TIMP: Tissue inhibitor of metalloproteinase; eNOS: Nitric oxide synthase; NO: Nitric oxide; ED: Endothelial dysfunction; ET-1: Endothelin-1; VSMCs: Vascular smooth muscle cells; ROS: Reactive oxygen species; TNF-a: Tumor necrosis factor-alpha; IL:  Interleukin This diagram has been created by the authors using Figdraw.

High levels of RC are strongly associated with arteriosclerosis. Excessive lipids and cholesterol bind to the arterial intima and accumulate in the arterial wall, causing oxidative and nitrosative stress that leads to chronic inflammation of the vascular wall and other mechanisms that increase vascular resistance, thereby causing or accelerating arteriosclerosis [7,37]. Yang et al.'s study found that RC was negatively correlated with flow-mediated vasodilation (FMD), representing endothelial function, and positively correlated with brachial-ankle pulse wave velocity, indicating that RC causes ED and subsequent arteriosclerosis [5,38,39]. In a study of patients with familial dysbetalipoproteinemia (FD) that increases RC levels, it was found that arterial wall and cell inflammation increased in FD patients. The results of this study indicate that RC affects arterial wall inflammation and circulating monocytes, leading to arteriosclerosis [40]. Studies have shown that RC can act on the vascular wall and induce the production of cytokines such as TNF-α, IL-6, and IL-1 [23]. This can cause inflammatory reactions and oxidative stress, leading to the formation of arteriosclerosis [38,39,41,42]. Taken together, these findings suggest that RC plays a crucial role in promoting arteriosclerosis through multiple mechanisms, including inducing endothelial dysfunction, inflammation, oxidative stress, and monocyte activation (Figure 2). These mechanisms highlight the importance of controlling RC levels to prevent or delay the development of arteriosclerosis.

Factors affecting RC levels and management and prevention strategies

Remnant cholesterol levels can be influenced by various factors, including genetic predisposition, lifestyles, drug therapy, and other environmental factors.

Impact of Genetic Factors on RC Levels

Previous genome-wide association studies (GWAS) on moderate to severe hypertriglyceridemia have found that the genetic basis is polygenic and can interact with environmental factors to affect the production and clearance of TG and VLDL [43]. Single nucleotide polymorphisms (SNPs) can have pleiotropic effects on lipid metabolism pathways, and these SNPs are mainly located on genes related to plasma TG levels, TGRLs, and CHD [44]. Genetic factors have a significant impact on lipid levels, and RC levels are also influenced by genetic factors. However, there are currently few studies on gene loci that independently affect RC level changes, and further research is needed to determine which genes specifically lead to CVD by affecting RC levels. This provides new possibilities for future RC reduction through gene regulation.

Impact of Diet and Lifestyle on RC Levels

Unhealthy lifestyle habits and poor dietary choices can have a significant impact on blood lipid levels. Elevated TG and RC levels are often caused by suboptimal lifestyle habits, such as consuming diets high in simple carbohydrates and/or saturated fats, excessive alcohol consumption, obesity, and leading a sedentary lifestyle [23]. The initial step recommended for managing hypertriglyceridemia is to adjust the diet and achieve weight loss. Dietary goals include avoiding highly refined carbohydrate foods, incorporating seafood in moderation, increasing intake of fiber-rich foods such as fruits, vegetables, and whole grains, limiting alcohol consumption, and substituting animal fat with unsaturated fat [45]. Thus, making healthy lifestyle changes and dietary choices, such as reducing intake of high-fat and high-sugar foods, following a high-fiber diet, achieving weight loss, quitting smoking, limiting alcohol consumption, and engaging in regular exercise, can effectively lower RC levels and prevent CVD.

Impact of Insulin Resistance on RC Levels

Insulin resistance can have a significant impact on RC metabolism. Impaired translocation of low-density lipoprotein receptor-related protein 1 (LRP1) from intracellular vesicles to the hepatic cytoplasmic membrane due to insulin resistance in the liver can result in a decreased clearance rate of TGRLs and increased RC levels. This weakened clearance of TGRLs by liver cells leads to RC accumulation [46]. Insulin resistance is one of the mechanisms behind high TG levels in obese patients with metabolic syndrome and type 2 diabetes. It can increase VLDL production by inhibiting apolipoprotein (apo)B degradation, leading to elevated TG and RC levels [23]. Therefore, managing insulin resistance through lifestyle changes, such as regular exercise and a healthy diet, and medication may help reduce RC levels and prevent or delay the development of CVD.

Impact of Drug Therapy on RC Levels

Drugs that lower TG levels are commonly used to reduce RC. The use of classic drugs and new therapies to lower TG levels can significantly reduce the plasma concentration of TGRLs and RC levels [45]. Classic lipid-lowering drugs, such as statins, fibric acid derivatives, and niacin, can all lower RC levels to some extent [28]. In recent years, new lipid-lowering treatments have been used in clinical practice. The European Atherosclerosis Society has proposed that lipid-lowering therapy can be achieved through mechanisms such as inhibiting lipoprotein production, reducing RC accumulation, stimulating fat breakdown, and enhancing RC clearance [45].

To inhibit lipoprotein production, antisense oligonucleotide inhibitors such as mipomersen and microsomal TG transfer protein inhibitors such as lomitapide can be used to inhibit apoB protein assembly, promote fatty acid oxidation, or reduce TG synthesis to reduce intracellular lipid overload [45]. Reducing RC accumulation can be achieved by administering cholesterol ester transfer protein (CETP) inhibitors alone or in combination with statins, which can lower LDL-apoB100 levels by increasing the apoB100 clearance rate [45]. Fibric acid derivatives are the main class of drugs used to stimulate fat breakdown. They promote lipolysis by increasing LPL activity and reducing apoC synthesis, thereby improving VLDL clearance efficiency [45]. Drugs that enhance RC clearance mainly include statins, proprotein convertase subtilisin/kexin type 9 (PCSK9) inhibitors, and angiopoietin-like protein 3 (ANGPTL3) inhibitors [45, 47]. In addition, apoC3 and apoA1 are also targets for lipid-lowering treatment [48].

When used alone or in combination, these drugs have varying degrees of effectiveness in reducing RC levels. However, regulating TG, TGRLs, and RC levels is complex, and developing drugs that lower one or more components of these lipoproteins while simultaneously reducing the risk of CVD is challenging and requires an improved understanding of the pathways that determine TG, TGRLs, and RC circulation. Further research is needed to discover other lipid-lowering drugs that are significant for the prevention and treatment of CVD.

## Conclusions

In conclusion, the relationship between RC, arteriosclerosis, and CVD is well-established, and lifestyle interventions and drug therapy have been shown to effectively lower RC levels. However, the lack of an established normal range or measurement standard for RC, as well as the need for further research to clarify its mechanism of action, highlights the importance of continued investigation in this area. Future studies on the treatment of RC and its relationship with CVD after treatment are also needed to advance our understanding and improve prevention and treatment strategies for arteriosclerosis and CVD.
